# Out-of-Hospital Cardiac Arrest As the Initial Presentation of Myxedema Coma

**DOI:** 10.7759/cureus.89399

**Published:** 2025-08-05

**Authors:** Mohamed Ibrahim, Hamzeh AL-Arqan, Phyo Maung

**Affiliations:** 1 Endocrinology, Diabetes and Metabolism, Blackpool Foundation Trust, Blackpool, GBR

**Keywords:** endocrinology and diabetes, hypothyroidism and bradycardia, hypothyroid myxedema coma, out of hospital cardiac arrest, thyroid disorder

## Abstract

Myxedema coma is a rare, life-threatening manifestation of severe hypothyroidism that typically presents in hospitalized patients with hypothermia, bradycardia, and altered mental status. In exceptionally rare instances, it may present with cardiac arrest as the initial manifestation.

We report a case involving a woman in her late 50s with obesity, obstructive sleep apnea, and previously undiagnosed hypothyroidism who experienced a witnessed out-of-hospital cardiac arrest. Following successful resuscitation, she was found to have profound hypothermia and bradycardia. Laboratory investigations revealed markedly decreased thyroid hormone levels with only modest elevation in thyroid-stimulating hormone. Her family reported a history of progressive lethargy, weight gain, cold intolerance, and confusion over several months. A prior elevated TSH had been documented one year earlier. Thyroid peroxidase antibodies were positive, while TSH receptor antibodies were negative.

She was treated with high-dose intravenous levothyroxine and liothyronine, in addition to intensive supportive care. Although her thyroid function improved biochemically and transient clinical improvement was noted, she developed aspiration pneumonia and progressed to septic shock, resulting in death on the fifth day of admission.

This case highlights the diagnostic challenge posed by myxedema coma, especially when TSH elevation is modest. Clinicians should maintain a high index of suspicion for endocrine causes of cardiac arrest and initiate prompt hormone replacement when indicated.

## Introduction

Myxedema coma is a rare but life-threatening endocrine emergency representing the extreme manifestation of untreated or undertreated hypothyroidism [1]. It is characterized by multiorgan dysfunction in the setting of severe hypothyroidism, typically occurring in older adults with long-standing thyroid hormone deficiency [2]. Despite advances in critical care and hormone replacement therapy, the condition carries a high mortality rate, reported to be between 30% and 60% in various studies [3,4].

Classically, patients present with hypothermia, bradycardia, hypotension, altered mental status, and features of myxedema such as dry skin and facial puffiness [1,5]. Precipitating factors often include infections, cardiovascular events, cold exposure, or sedative use [2,6]. However, the clinical presentation is often subtle or atypical, leading to delayed recognition [3]. Moreover, biochemical parameters such as thyroid-stimulating hormone (TSH) may not accurately reflect the clinical severity. In particular, some patients present with only modest elevations in TSH, especially in central or mixed hypothyroidism or during critical illness [7].

This case is unusual in that the initial presentation was a witnessed out-of-hospital cardiac arrest, with subsequent biochemical evidence of profound hypothyroidism but only a modest TSH elevation. Such presentations are exceedingly rare [8] and underscore the importance of maintaining a high index of suspicion for endocrine causes of cardiac arrest. This report highlights the diagnostic challenges associated with myxedema coma, particularly in atypical presentations, and emphasizes the importance of early thyroid hormone replacement alongside intensive supportive care [1,5].

## Case presentation

A woman in her late 50s with a background of obesity, obstructive sleep apnea managed with CPAP, and a history of smoking experienced a witnessed out-of-hospital cardiac arrest. Cardiopulmonary resuscitation was promptly initiated by a bystander, and return of spontaneous circulation was achieved by paramedics. Upon arrival at the emergency department, she was intubated and mechanically ventilated.

**Figure 1:**
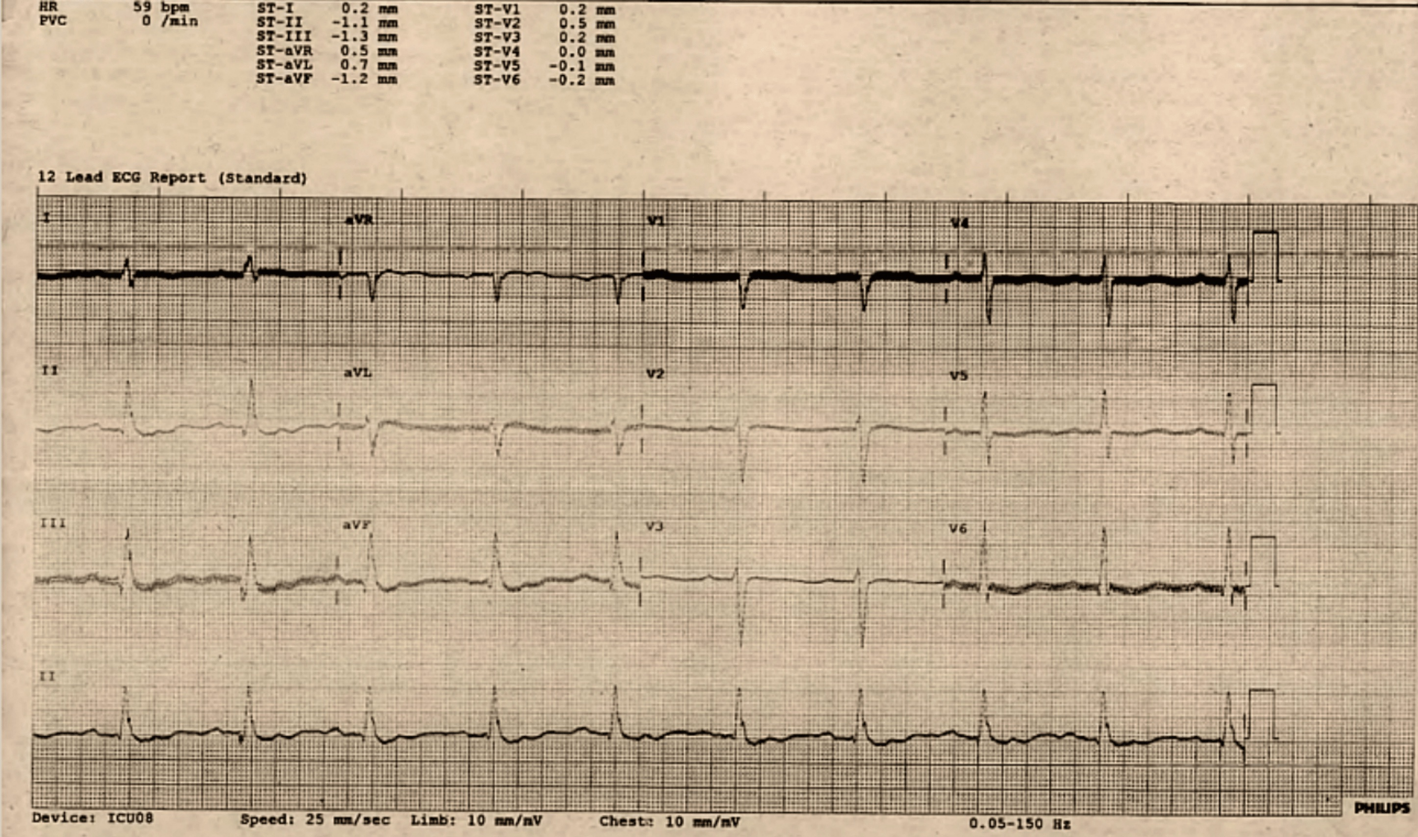
12-lead ECG showing sinus bradycardia (~55bpm) in the setting of severe hypothyroidism and myxedema coma. Bradycardia persisted despite inotropic support.

On examination, she was profoundly hypothermic and bradycardic, with additional clinical features suggestive of hypothyroidism, including periorbital puffiness and dry, coarse skin. An electrocardiogram (ECG) performed shortly after resuscitation revealed persistent sinus bradycardia despite ongoing inotropic support (Figure 1), consistent with severe metabolic suppression [1].

Family members reported that the patient had been experiencing progressive lethargy, cold intolerance, weight gain, and cognitive decline over several months. Initial laboratory investigations (Table 1) showed markedly decreased free T4 and free T3 levels, with a modestly elevated TSH. Cortisol, follicle-stimulating hormone (FSH), and luteinizing hormone (LH) were within normal limits. Autoimmune testing revealed significantly elevated thyroid peroxidase antibodies and negative TSH receptor antibodies.

**Table 1:** Laboratory Investigations on Admission

Test	Result	Reference Range	Interpretation
Free T4 (pmol/L)	<1.3	12.0 – 22.0	Severely decreased
Free T3 (pmol/L)	<0.3	3.1 – 6.8	Severely decreased
TSH (mIU/L)	23.9	0.4 – 4.0	Moderately elevated
Cortisol (nmol/L)	823	140 – 690	Increased
FSH (IU/L)	18.2	Postmenopausal: 25.8 – 134.8	Decreased
LH (IU/L)	1.2	Postmenopausal: 7.7 – 58.5	Decreased
Thyroid Peroxidase Ab (IU/mL)	554	<35	Significantly elevated
TSH Receptor Ab	Negative	Negative	Negative

Based on the clinical presentation, biochemical findings, and history, a diagnosis of myxedema coma was established. The patient was treated with high-dose intravenous levothyroxine and liothyronine, alongside comprehensive supportive care in the intensive care unit. Thyroid function subsequently improved, as reflected by normalized free T3 and free T4 levels and a decreased TSH (Table 1). Although the patient initially showed transient clinical improvement, she developed aspiration pneumonia, which progressed to septic shock. Despite maximal supportive therapy, she died on the fifth day of admission [6].

Informed written consent was obtained from the patient’s next of kin for the open-access publication of this case report.

## Discussion

Myxedema coma is a rare, life-threatening endocrine emergency resulting from severe and prolonged hypothyroidism, most commonly due to autoimmune thyroiditis such as Hashimoto’s disease [1,2]. It is associated with a high mortality rate and typically presents in elderly patients with hypothermia, altered mental status, and multiorgan dysfunction [3,4]. However, as demonstrated in this case, presentation can be dramatic and atypical, including events such as out-of-hospital cardiac arrest [5].

A notable feature in this case was the biochemical discordance: while free T3 and free T4 levels were severely decreased, the TSH elevation was only moderate. This highlights a diagnostic challenge seen in acute decompensated hypothyroidism, where TSH may not rise proportionally to the severity of hormone deficiency [6]. This discordance may be particularly evident in patients with long-standing hypothyroidism, central hypothyroidism, or suppression of the hypothalamic-pituitary-thyroid axis due to critical illness [7,8]. Thus, clinical judgment-guided by free hormone levels and physical findings-remains paramount in diagnosing myxedema coma.

While the diagnosis is clinical, supported by typical laboratory findings, diagnostic tools such as a scoring system may assist in ambiguous presentations [9]. This case fulfilled both clinical and biochemical criteria, including altered sensorium, hypothermia, bradycardia, and markedly suppressed thyroid hormone levels in the context of known risk factors.

Treatment involves urgent thyroid hormone replacement-typically with intravenous levothyroxine and/or liothyronine-alongside supportive care in an intensive care setting [4,10]. Empiric glucocorticoids are often administered initially to cover potential adrenal insufficiency until cortisol results are available [3]. In this patient, thyroid function improved with therapy, but her course was complicated by aspiration pneumonia and subsequent septic shock, ultimately leading to death despite maximal intervention [5].

This case underscores the importance of maintaining a high index of suspicion for myxedema coma in patients presenting with unexplained bradycardia, hypothermia, or cardiac arrest-especially when there are supporting features or a known history of thyroid dysfunction [2,6]. Early recognition and prompt initiation of hormone therapy may significantly alter outcomes [10].

## Conclusions

Myxedema coma is a rare but life-threatening endocrine emergency that may present atypically, including with sudden cardiac arrest. This case highlights the need for a high index of suspicion, especially when clinical signs suggest hypothyroidism-even in the absence of markedly elevated TSH. In acute decompensated states, free thyroid hormone levels and the overall clinical picture offer more reliable diagnostic guidance.

Timely initiation of intravenous thyroid hormone therapy, alongside intensive supportive care, remains the cornerstone of management. Early recognition-guided by subtle historical clues and clinical acumen-may be life-saving in this otherwise high-mortality condition.
